# mSphere of Influence: Whole-Genome Sequencing, a Vital Tool for the Interruption of Nosocomial Transmission

**DOI:** 10.1128/mSphere.00230-21

**Published:** 2021-04-28

**Authors:** Ahmed Babiker

**Affiliations:** a Emory University School of Medicine, Department of Medicine, Division of Infectious Diseases, Atlanta, Georgia, USA; b Emory University School of Medicine, Department of Pathology and Laboratory Medicine, Atlanta, Georgia, USA

**Keywords:** whole-genome sequencing, outbreak investigation, infection prevention, carbapenem-resistant *Klebsiella pneumoniae*, KPC

## Abstract

Ahmed Babiker’s work focuses on the clinical and genomic epidemiology of multidrug-resistant health care-associated pathogens and other high-consequence pathogens. In this mSphere of Influence article, he reflects on how the paper “Tracking a Hospital Outbreak of Carbapenem-Resistant Klebsiella pneumoniae with Whole-Genome Sequencing” by Evan S. Snitkin et al. (Sci Transl Med 4:148ra116, 2012, https://doi.org/10.1126/scitranslmed.3004129) impacted his thinking on the use of whole-genome sequencing for nosocomial transmission investigation.

## Abstract (precis)

Ahmed Babiker’s work focuses on the clinical and genomic epidemiology of multidrug-resistant health care-associated pathogens and other high-consequence pathogens. In this mSphere of Influence article, he reflects on how the paper “Tracking a Hospital Outbreak of Carbapenem-Resistant Klebsiella pneumoniae with Whole-Genome Sequencing” by author Evan S.

## COMMENTARY

In 2011, the U.S. National Institutes of Health (NIH) Clinical Center experienced a prolonged outbreak of carbapenem-resistant Klebsiella pneumoniae (CRKP) among 18 patients, with 6 attributable deaths. In the paper “Tracking a Hospital Outbreak of Carbapenem-Resistant Klebsiella pneumoniae with Whole-Genome Sequencing” (1), Evan S. Snitkin et al. apply genomic approaches to unravel the outbreak and decipher transmission events. The paper was among the first to highlight asymptomatic colonization and environmental persistence as major elements in nosocomial transmission pathways. For myself, the true lesson learned was the impact that real-time genomics might have on a hospital outbreak investigation. Reading this paper a few years postpublication as an undifferentiated first-year infectious disease fellow motivated me to seek out mentorship in genomic data analysis and interpretation. This pivot had a major impact on developing my research focus and my career path thus far.

Snitkin et al. applied an integrated genomic and epidemiological analysis to track a prolonged outbreak of CRKP at their institution. Inferring potential transmission events solely on the basis of epidemiologic data proved challenging due to the extensive overlap of patients in hospital wards, so whole-genome sequencing (WGS) was performed to gain further insight. The authors sequenced seven isolates from the suspected index patient. This revealed a total of seven single-nucleotide variants (SNVs) among the isolates. Identification of this genetic heterogeneity within the index patient’s isolates proved invaluable in reconstructing transmission pathways. During the course of the outbreak, three rounds of rectal surveillance cultures were performed on all hospital patients to identify asymptomatically colonized patients. Sequencing of a single CRKP isolate from each of the remaining 17 identified patients allowed investigators to group isolates on the basis of shared variants into three distinct clusters. Putative transmission maps were constructed using detailed patient location and genomic data, with consideration given to the possibility of silent (undetected) colonization. Notably, transmission maps generated on the basis of either patient trace or genomic data alone differed, highlighting the synergistic power of combining both data types. Ultimately, the application of WGS allowed the authors to come to the following key conclusions. First, the outbreak was likely monoclonal and introduced by the index patient. This is despite a 3-week interval between the index case and the identification of subsequent cases. Second, three independent transmission events occurred from at least two anatomical sites from the index patient, leading to at least two major clusters. Third, CRKP environmental stability facilitated transmission to at least one patient via a contaminated ventilator. These findings underlined how the precision offered through real-time sequencing may yield actionable data to aid infection prevention efforts. Since the publication of the original paper, Snitkin et al. have applied this approach on a larger scale to investigate a U.S. regional outbreak of CRKP (2). Here, authors integrated genomic and interfacility patient transfer data. Once again, genomic analysis produced a high-resolution transmission network that assigned directionality to regional transmission events and discriminated between intra- and interfacility transmission when epidemiologic data were ambiguous or misleading (2).

This paper and other contemporary landmark papers (3, 4) convinced me that WGS should be integrated into routine hospital infection prevention workflows. With continued improvements in accessibility, turnaround time, cost, and access to user-friendly bioinformatic tools (5), genomic data can be generated in a clinically relevant and actionable time frame. Furthermore, my experience with the Microbial Genomic Epidemiology Laboratory (MiGEL) at the University of Pittsburgh has shown me the clear need for a shift in our current paradigm for hospital outbreak detection. Rather than employing WGS in a reactive manner for outbreak resolution, prospective routine sequencing should be applied for characterization of the local epidemiology of key health care-associated pathogens and real-time surveillance. This can lead to expedited outbreak detection and response. Especially in the setting of high numbers of epidemiologically unrelated cases (e.g., infections by vancomycin-resistant enterococcus, methicillin-resistant Staphylococcus aureus [MRSA], and *Clostridioides* [formally *Clostridium*] *difficile*), which may create substantial background noise (6). The utility of such a paradigm shift has been demonstrated through an innovative surveillance project, titled Enhanced Detection System for Hospital-Acquired Transmission (EDS-HAT), implemented by the MiGEL group (6, 7). EDS-HAT combines prospective WGS of selected nosocomial pathogens and a machine-learning automated data-mining algorithm of the electronic medical record (EMR) to determine routes of transmission among clusters of phylogenetically related pathogens. This novel approach allows for detection of transmission clusters beyond those identified through geo-spatial clustering. Prospective sequencing during the 1-year developmental and validation phase of EDS-HAT uncovered numerous clusters undetected through routine surveillance. Epidemiological investigation of these clusters confirmed novel and previously undescribed transmission routes which had been identified by the EMR data-mining algorithm (6, 7). A similar prospective integrated epidemiological and phylogenetic analysis applied at the population level provided a comprehensive picture of MRSA transmission in eastern England (8). Through this approach, authors concluded that MRSA transmission was driven by a number of unrecognized outbreaks. Moreover, they identified the critical role played by persistent carriers who spread MRSA in multiple wards by means of complex health care pathways, likely through environmental contamination and/or colonized health care workers.

Analysis and data such as that provided by Snitkin et al. have continued to enrich how we view nosocomial transmission. In particular, it has elucidated the role of asymptomatic colonization and environmental reservoirs (9). This understanding, as well as advances in microbial therapeutics for multidrug-resistant organism (MDRO) decolonization, spurred me to join a group of investigators at Emory who intend to apply integrated genomic and metagenomic methods and environmental sampling to study innovative interventions to reduce nosocomial transmission (10). With refinement of sequencing techniques, methodology, and accessibility, I am optimistic that whole-genome sequencing will no longer be viewed as an adjunct tool for outbreak resolution but rather as a cornerstone of hospital pathogen surveillance.
